# Targeted vaccination campaigns of teenagers after two clusters of B invasive meningococcal disease in Brittany, France, 2017

**DOI:** 10.1186/s12889-020-09487-7

**Published:** 2020-09-10

**Authors:** Mathilde Pivette, Muhamed-Kheir Taha, Anne-Sophie Barret, Elisabeth Polard, Marie-Bernadette Hautier, Jean-Benoît Dufour, Marlène Faisant, Lisa Antoinette King, Denise Antona, Daniel Levy-Bruhl, Hélène Tillaut, Alexandre Scanff, Camille Morival, José-Hector Aranda Grau, Pierre Guillaumot, Bertrand Gagnière

**Affiliations:** 1 Santé publique France, French national public health agency, Direction des régions, Cellule Bretagne, Rennes, France; 2 Institut Pasteur, National Reference Center for Meningococci, Paris, France; 3 Santé publique France, French national public health agency, Direction des maladies infectieuses, Saint-Maurice, France; 4 Pharmacovigilance, Pharmacoepidemiology and Drug Information Center, Rennes University Hospital, Rennes, France; 5 Direction académique de l’Education Nationale, School Health services, Saint-Brieuc, France; 6 Agence régionale de santé Bretagne, Regional health agency, Rennes, France

**Keywords:** Serogroup B meningitis, Vaccination campaign, Adolescent, France

## Abstract

**Background:**

In December 2016, three cases of serogroup B invasive meningococcal disease, including two children from the same middle school (11 to 15 years old pupils), occurred in the department (administrative district) Côtes-d’Armor (Brittany, France). They were infected by a rare strain (B:P1.7–2,4:F5–9:cc162), covered by the 4CMenB vaccine (Bexsero®). Four months later, two cases due to the same strain occurred in a high school in the same area (15 to 19 years old students). In accordance with French recommendations, vaccination was proposed to students of both schools and to all individuals aged 11–19 years living or studying in the hyperendemic area. We describe these vaccination campaigns, from the alert to the impact evaluation.

**Methods:**

The target population included 8884 people: 579 in the middle school, 2007 in the high school and 6298 in the community. In both schools, vaccination sessions were organized directly on site. In the community, teenagers were vaccinated by general practitioners. The vaccination campaign took place from May to October 2017. An active pharmacovigilance follow-up was set up to document adverse effects of the vaccine.

**Results:**

Considering the whole target population, the vaccination coverage was estimated at 43% for 1 dose and 34% for 2 doses. Higher vaccination coverage was observed in the schools (79% in the middle school and 42% in the high school for 2 doses) than in the community (27% for 2 doses). The reported adverse effects were consistent with the safety profile of the vaccine and no severe adverse effect was reported.

**Conclusions:**

This vaccination campaign was the third one implemented with Bexsero® in France and constitutes a reproducible approach for future targeted vaccination campaigns. No additional cases of the same strain have occurred since the end of the campaigns in the area.

## Background

Invasive meningococcal disease (IMD) is a severe bacterial infection caused by *Neisseria meningitidis*, which can lead to sequelae and death. Meningococcal bacteria are transmitted through respiratory or throat secretions and the incubation period ranges from 2 to 10 days. In France, annual incidence rates of IMD varied between 0.7 to 1.3 cases per 100,000 inhabitants between 2006 and 2016 [1]. Children under the age of 5 and young adults (15–24 years-old) are the most affected by the disease [2]. The serogroup B is predominant in France, accounting for 51.6% of the cases in 2016, with a case fatality rate of 8% [3].

Higher meningococcal carriage rates were reported in teenagers in previous studies. Carriage prevalence was estimated to increase throughout childhood from 5% in infants to 8% in 10-year olds and peaking at 24% in 19-year olds before decreasing through adulthood (from 13% at 30 years old to 8% at 50 years old) [4].

When an IMD case occurs, a chemoprophylaxis is systematically proposed to all close contacts exposed in the ten days preceding the IMD onset, in accordance with French recommendations [5].

The majority of serogroup B IMD cases are sporadic and outbreaks are rare [6–8]. A vaccine against IMD serogroup B (4CMenB/ Bexsero®) was licensed in Europe in 2013. The vaccination scheme of Bexsero® for teenagers and adults is two doses administered at an interval of at least one month. The vaccine is not included in the French routine immunization program. It is only recommended for at-risk individuals (eg. people with asplania, complement deficiency) or in specific epidemiological situations (clusters, outbreaks or hyperendemic situations) after a risk assessment by health authorities [5, 9].

In December 2016, three cases of serogroup B IMD, including two children from the same middle school, occurred in the district Côtes-d’Armor (Brittany, France). They were all infected by the same strain which was covered by the Bexsero® vaccine on the basis of the expression of PorA P1.4 which is a perfect match with one of the vaccine components [10]. A vaccination campaign was implemented in the school. Four months later, two additional cases due to the same strain occurred in a high school of the same area. Overall, five cases occurred within a 4 month period. Vaccination was then proposed to the students of the high school and to all individuals aged 11–19 years living or studying (15 schools) in the hyperendemic area.

Since the vaccine was licensed in 2013, only two vaccination campaigns with Bexsero® had been previously organized in France [8, 11]. We describe here the vaccination campaigns implemented following the detection of two clusters of serogroup B IMD in Brittany, from the alert to the impact evaluation.

## Methods

### IMD surveillance

In France, IMD is a mandatory notifiable disease. All cases of IMD are notified to the regional health agency (RHA) that collects information on the cases (clinical data, sociodemographic characteristics and activities during the 10 days before symptom onset) and identifies close contacts in order to recommend prophylactic antibiotics (and vaccination for serogroups ACWY) [9]. Notification forms are then transmitted to the French public health agency which is charge of epidemiological surveillance.

For all cases, meningococcal cultured isolates or primary clinical samples are sent to the National Reference Center for meningococci for full characterization and typing. The grouping and genotyping uses multilocus sequence typing (MLST), which determines the sequence type and the clonal complex (cc) of isolates. The typing data are expressed as a combination of group, variable VR1 and VR2 of PorA, variable region of the protein FetA and clonal complex.

Furthermore, because of French vaccination strategies against B IMD, serogroup B isolates corresponding to clusters are also investigated for coverage by the Bexsero® vaccine. In general, an isolate is covered if the sequence of PorA P1.4 antigen is detected and/or if the expression level of at least one of NHBA, fHbp or NadA antigens is higher than the protective bactericidal threshold, as determined by the Meningococcal Antigen Typing System (MATS) [10]. MATS can be applied when culture isolates are available. In this study, the diagnosis was confirmed by PCR only and no cultured isolates were available.

### Epidemiological analysis and decision-making

Following each cluster of serogroup B IMD cases, epidemiological investigations were conducted by the French national public health agency (Santé publique France) to describe the situation, assess the risk and guide control measures. The study area was defined as the smallest geographical area covering the place of study or of residence of cases. Incidence rates of serogroup B IMD in the total population and by age group were calculated in the defined area and compared with rates observed in mainland France (excluding the affected department). Risk assessment and decision making were performed using criteria defined in French directives as follow [9]:

- Cluster: occurrence of ≥2 cases caused by identical or indistinguishable strains, in the same community or social group within 3 months.
- Hyperendemic: occurrence of ≥4 cases caused by identical or indistinguishable strains with an incidence rate ≥ 3 cases per 100,000 inhabitants within 52 weeks in a defined area.
- Epidemic: occurrence of ≥3 cases caused by identical or indistinguishable strains, in the same community without direct contact, with an attack rate ≥ 10 cases per 100,000 inhabitants within 3 months.

**Fig. 1:**
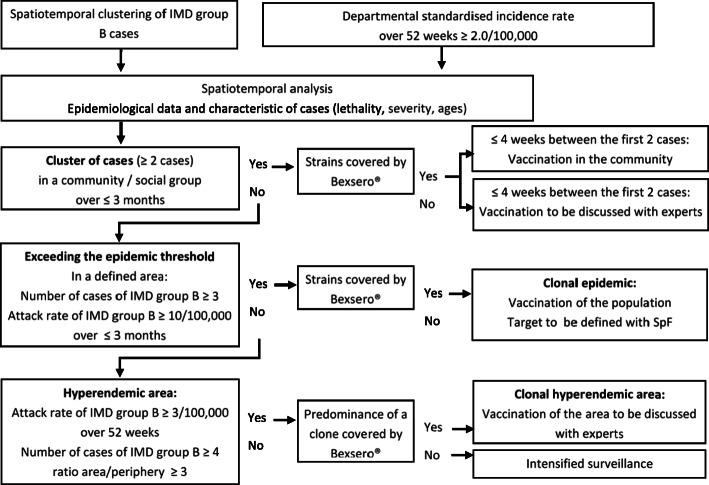
Algorithm for decision-making for vaccination with Bexsero® [8]

A multidisciplinary expert committee was convened to discuss whether vaccination was appropriate, taking into account the algorithm for vaccination decision-making defined in the national guidelines (Fig. 1).

### Vaccination coverage estimates

In the middle school (11 to 15 years pupils) and in the high school (15 to 19 years old students), vaccination sessions were organized directly on site for each dose. The vaccination coverage was calculated in real-time after each vaccination session as the ratio of the number of vaccinated children to the number of children in the school.

In the community (11 to 19 years old individuals living or studying in the area), two sources of data were used to assess vaccination coverage during the campaign.

The total number of vaccine doses distributed to pharmacies was obtained from wholesale distributors. These distribution data were exhaustive and were transmitted regularly by wholesale distributors to the RHA during the campaign.

To estimate the proportions of dose 1 and dose 2 that were distributed, we used delivery data from a sample of 18 pharmacies in the area which received 62% of the total vaccines distributed by wholesale distributors during the campaign. A medical prescription was required for vaccination and the pharmacies registered the doses delivered to their patients in their information system which is linked with French health insurance system. They can therefore differentiate between dose 1 and dose 2 for each patient. In the middle and at the end of the campaign, each pharmacy transmitted to the RHA their numbers of first and second doses delivered.

We hypothesized that the proportions of doses 1 and 2 in the sample of pharmacies were not different from that of the other pharmacies and applied these proportions to all doses distributed by wholesale distributors. The vaccination coverage was calculated for each dose as the ratio of the estimated number of doses 1 and doses 2 delivered by pharmacies to the number of persons in the target population.

### Pharmacovigilance follow-up

The Regional Pharmacovigilance Center (RPC) set up a follow-up to document adverse effects of the vaccine using a specific questionnaire. The information collected in the questionnaire was administrative and about adverse effects: type of adverse effects (local reactions, systemic reactions), description of the effects, date of onset after injection, and duration of the effect. The child or his parents received the document just after vaccination and for each dose.

In the middle school, a reinforced pharmacovigilance follow-up was set up in order to complete the safety profile of the vaccine as its use was recent in France in relation with the National Reference Center for Meningococci. Parents were asked to complete and send the document to the RPC within 15 days after injection whether or not adverse effects occurred.

Otherwise, in the high school and in the community, parents and general practitioners were asked to complete and sent the document within 15 days after injection only in case of adverse effects, as usual in pharmacovigilance follow-up.

### Ethics

This investigation was implemented in compliance with the authorization delivered to the French national public health agency (Santé publique France) by the French data protection authority (CNIL) to process personal health data in order to prevent, alert or monitor an epidemiological outbreaks (authorization 341,194 V42). All the pharmacovigilance reports were registered in the French Pharmacovigilance Database.

## Results

### First vaccination campaign: January to march 2017

#### Description of the cases

Two cases of serogroup B IMD occurred in December 2016 within 24 h in two children who were enrolled in two different classes of the same middle school in the department Côtes-d’Armor (11 to 15 years old pupils, 579 pupils). They both attended the school during the 10 days preceding the onset of symptoms but no direct contact between them was reported. The two cases were defined as co-primary as they occurred in the same community in less than 24 h, in accordance with the French guideline on investigation of cluster of IMD [8]. They were thus considered as one primary episode for epidemiological analysis. Another case occurred one week later in a teenager living 30 km from the middle school. No epidemiological link between this case and the two others was identified. Molecular typing showed that the three cases were infected by isolates with the same genotypic formula: B: P1.7–2, 4:F5–9:cc162, which was covered by the vaccine Bexsero® on the basis of the presence of the sequence of the PorA P1.4 antigen. The three cases recovered completely.

#### Epidemiological analysis

In the smallest geographical area covering the place of study of the 2 first cases and place of residence of the third one, the incidence rate in the three preceding months was 1.9 per 100,000 inhabitants. Criteria were not met to consider the area as hyperendemic nor epidemic. The situation was treated as a cluster of two cases of serogroup B IMD of the same strain, covered by the vaccine Bexsero®, occurring in the same community, the school, within less than 4 weeks.

#### Decision-making

In accordance with national guidelines, the expert group decided to propose vaccination to both children and adults of the school because the at-risk community was the school. The target population included 579 pupils aged between 11 and 15 years old and 86 adults aged between 22 and 61 years old.

#### Organization of the campaign

A public meeting was held by the RHA in January to provide parents information on meningococcal infections, the epidemiological situation, the vaccine and its adverse effects. The RHA organized vaccination sessions directly in the school during two days in January 2017 for the first dose and two days in March 2017 for the second dose. Vaccines doses were distributed by the local hospital to the school. Children had to present a parental authorization to be vaccinated, and they had a medical consultation on site before vaccination. Medical doctors, nurses, epidemiologists and civil protection volunteers were involved in the vaccination sessions.

### Second vaccination campaign: May to October 2017

#### Description of the cases

Four months later, in April, two additional cases of serogroup B IMD occurred within 3 days in two teenagers, who were enrolled in a high school (15 to 19 years old students, 2007 students), approximately 30 km from the school of the first two cases, and 10 km from the third case. No epidemiological links were identified between these two cases and the first three cases.

They did not attend the school during the 10 days preceding the onset of symptoms as it was school holidays. No direct contact between the two cases during this period had occurred, but they had contacts with common friends. Isolates from both cases had the same genotypic formula as the cases of December B: P1.7–2, 4:F5–9:cc162. Both cases recovered completely.

#### Epidemiological analysis

**Fig. 2:**
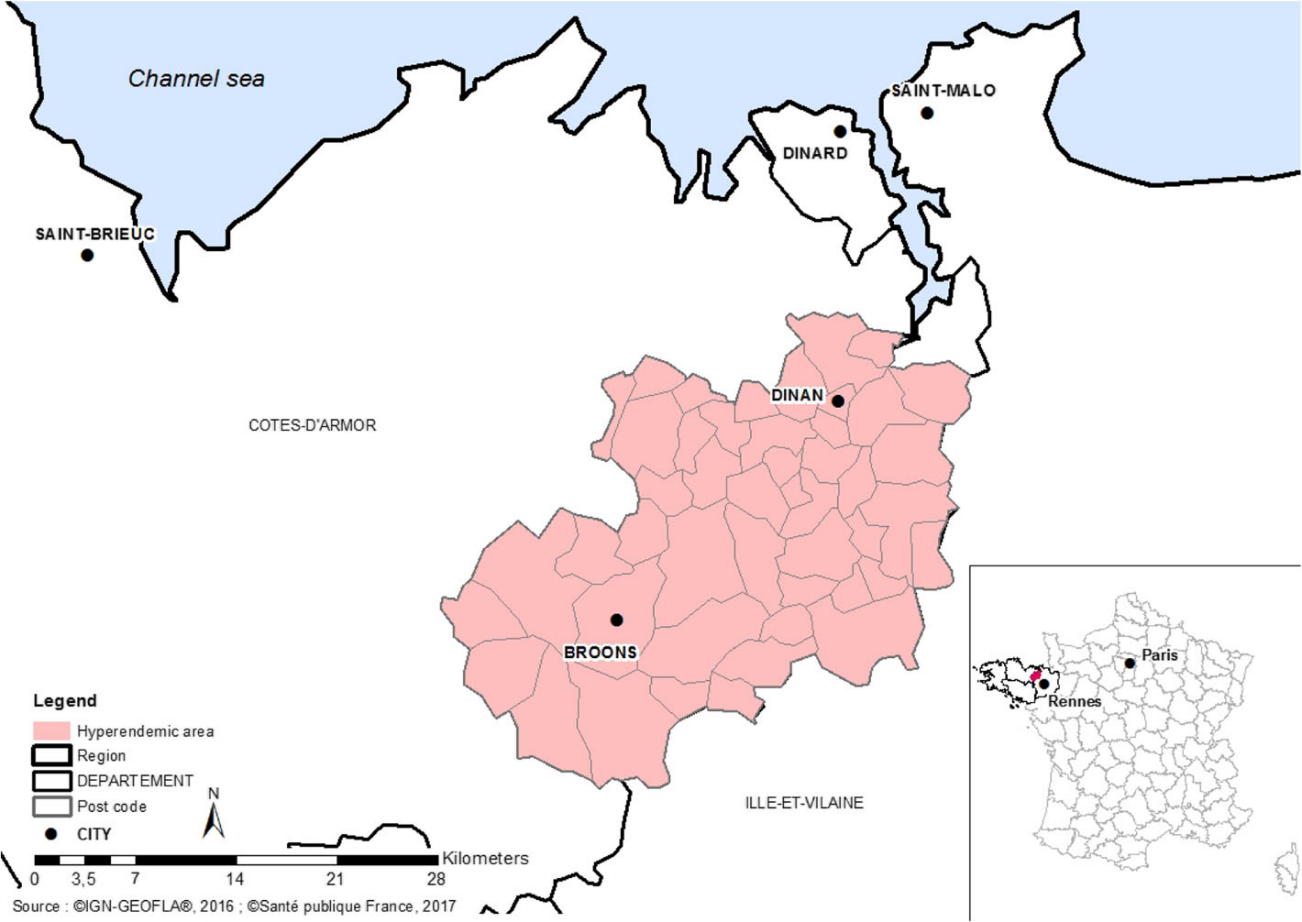
Localization of the hyperendemic area, Côtes-d’Armor, Brittany. (Sources:©IGN-GEOFLA®, 2016;©Santé publique France, 2017). Map was created using ArcGIS® 10.2.2 software by Esri, under license

The smallest area covering the place of residence or study of the 5 cases confirmed as serogroup B IMD cc 162 (3 cases in December and 2 cases in April) comprised 47 municipalities (either cities or villages), including 62,917 inhabitants (Fig. 2). The B IMD cc 162 incidence rate in this area between May 2016 and April 2017 was 6.4 per 100,000 inhabitants, versus 0.3 in the rest of mainland France for B IMD (excluding the department Côtes-d’Armor). This incidence rate was thus above the hyperendemic threshold defined for serogroup B IMD (3 per 100,000 inhabitants). The attack rate in the 10–19 age group was 57.2 cases per 100,000 (Table 1). The situation was a cluster of two cases in a high school and a hyperendemic situation in the area of 47 municipalities.

**Table 1:** Incidence rates of serogroup B cc162 IMD in the hyperendemic area and of serogroup B IMD in mainland France without the department Côtes-d’Armor, during 52 weeks (21/04/2016 to 20/04/2017)

	Hyperendemic area			Mainland France (without Côtes-d’Armor)		
Age group	Population	Number of B cc162 IMD cases	Incidence/100000 inhabitants	Population	Number of B IMD cases	Incidence/100000 inhabitants
0–4	3797	0	–	3,805,273	78	2.0
5–9	4057	0	–	3,890,742	18	0.5
10–14	3806	1*	26.3	3,884,039	11	0.3
15–19	3188	3	94.1	3,795,725	22	0.6
20–49	21,313	0	–	24,225,556	45	0.2
≥50	26,756	0	–	23,499,232	38	0.2
10–19	6994	4	57.2	7,679,764	33	0.3
All ages	62,917	4	6.4	63,100,567	212	0.3

*The two co-primary cases were considered as one episode for epidemiological analyses

#### Decision-making

In accordance with national guidelines [5], the expert committee decided to propose vaccination to students of the high school and to the community defined as all individuals aged 11–19 years living or studying in one of the 15 other schools in the hyperendemic area. This population was targeted first because all cases had occurred in this age group (11–19 years), second because the occurrence of two clusters caused by the same strain in two different schools without identified links suggested the circulation of the bacteria among adolescents in the area, and third because middle school starts from 11 years old.

The target population included 2007 students in the high school and 6298 individuals in the community (INSEE RP 2014/ Academic Direction of the National Education system).

#### Organization of the campaign

In the high school, a public meeting was held for parents in April. The vaccination sessions in the high school were organized directly on site during two days in May 2017 for the first dose and two days in June 2017 for the second dose. The organization was similar to that of the first campaign.

In the community, people had to consult their general practitioners (GPs) to obtain a prescription of the two-dose vaccine Bexsero®. Doses were delivered by pharmacists at an interval of at least one month and administered by GPs or private nurses. The vaccine was fully reimbursed by the health care system for the target population. As the vaccine is not included in the national immunization program, pharmacies generally have no stock. Therefore, the RHA organized the supply of vaccines to pharmacies through wholesale distributors.

No individual invitation was sent to the targeted individuals in the community but a communication campaign was set up in the area. The RHA organized meetings for parents in the other schools of the hyperendemic area in May. Information about the campaign was also transmitted to health professionals (GPs, pharmacists, private nurses) and a meeting with them was organized. The mayors of the 47 municipalities of the hyperendemic area were informed and a press release was issued.

A toll-free number was set up during one week in May at the beginning of the campaign by the French health reserve and the RHA to answer questions from health professionals and the public. One month later, mid-June, information about the second dose was transmitted to health professionals and to the press. The vaccination campaign in the community started in May 2017 and ended in October 2017.

### Vaccination coverage estimates

In the middle school, the vaccination coverage in children was 84% for 1 dose and 79% for 2 doses. The vaccination coverage in adults was 60% for 1 dose and 41% for 2 doses.

In the high school, the vaccination coverage was lower than in the middle school, 56% for 1 dose and 42% for two doses.

In the community, the wholesale distributors distributed 3949 doses of vaccines to seventy-three pharmacies between May and October 2017. In the sample of 18 pharmacies which received 62% of the total vaccines distributed, 57% of the doses delivered were first doses and 43% were second doses. After applying this proportion to all doses distributed by wholesale distributors, the number of first doses was estimated at 2237 and the number of second doses at 1712. The vaccination coverage in the community was then estimated to be 36% for 1 dose and 27% for 2 doses.

Considering the whole target population of 11–19 years-old living or studying in the hyperendemic area (8884 persons), the vaccination coverage was estimated at 43% for 1 dose (3846 doses) and 34% for 2 doses (3011 doses) (Table 2).

**Table 2:** Vaccination coverage estimates in the 11–19 year-olds living or studying in the hyperendemic area, according to the place of vaccination

	Target population	1 dose		2 doses	
		Vaccinated population	Vaccination coverage	Vaccinated population	Vaccination coverage
**Middle school**	579	484	**84%**	458	**79%**
**High school**	2007	1125	**56%**	841	**42%**
**Community**	6298	2237	**36% [34–37]**	1712	**27% [26–29]**
**TOTAL**	8884	3846	**43% [42–44]**	3011	**34% [33–35]**

*[*] *confidence interval 95%*

### Pharmacovigilance follow-up

Overall, 1374 adverse effects (AE) were reported concerning 412 individuals.

The notification rate of adverse effects (number of notifications per 100 vaccines injected) was 31.2% for the middle school (321 notifications/1029 vaccines injected). This notification rate was 3.7% in the second campaign, high school and community considered together (218 notifications/5915 vaccines injected).

No AE was considered severe according to the World Health Organization criteria of severity. Two effects were medically significant (vestibular neuritis, leg paresis) with favorable outcome, a link with vaccination could not be excluded. The majority of AE were local reactions (75%) and less frequently systemic reactions, mainly fever and headache (10%), with favorable outcome within a week. Some individuals (127) presented AE after each dose, usually similar in nature and intensity.

## Discussion

Between December 2016 and April 2017, a hyperendemic situation of meningitis due to a rare strain (B: P1.7–2, 4:F5–9:cc162) was observed in a small area in northwest of Brittany, with two clusters of two cases each and one additional isolated case. Cases only occurred in adolescents who were therefore the age group targeted by the vaccination campaign. No additional cases of the same strain occurred since the end of the campaigns in the area (up to April 2020). The last case of this strain reported in Brittany was in April 2017 in the Ille-et-Vilaine department.

The Brittany region was characterized by a high incidence rate of meningitis B over the period 2006–2016 (0.87 per 100,000 inhabitants, 1.6 times higher than that observed in mainland France). Strains with genotype B: P1.7–2, 4:F5–9:cc162, were rare in France between 2011 and 2015 and represented only 4.5% of all the circulating B characterized genotypes (41 cases including 2 in Brittany) [2]. Between January 1, 2016 and April 30, 2017, 18 cases with this genotype B: P1.7–2, 4:F5–9:cc162 occurred in France, of which 10 were detected in Brittany in three different departments. Apart from the clusters detected in Côte-d’Armor, there was no clustering of cases nor epidemiological links between the cases reported in Brittany. These data suggested the recent emergence of this strain in Brittany with the potential to cause local hyperendemic situations.

The multicomponent group B meningococcal vaccine Bexsero® was first licensed in Europe in 2013 and is now licensed for use in several countries worldwide. It has been included in national immunization program for infants in some countries (UK, Ireland, Italy, Andorra, San Marino, Lithuania, Czechia) [12–14]. The Bexsero® does not seem to have an effect on carriage [15], with an unfavorable cost-effectiveness ratio [16]. In France, these considerations, did not support introduction of Bexsero® into the universal vaccination program. Currently, it is only recommended in at-risk individuals and for specific epidemiological situations (outbreak, hyperendemic situation) [5]. The relevance and the efficiency of the strategy aimed at detecting local clusters compared to universal vaccination cannot be evaluated in the framework of our investigation. However, the implementation of such large campaigns provide useful information to policy makers and public health agencies that may face similar epidemiological situations.

In France, the vaccination campaign in Côtes-d’Armor was only the third one implemented with Bexsero® since 2013. In late 2013, the vaccine Bexsero® was recommended to control an hyperdendemic situation caused by serogroup B cc16 strain in the regions of Normandy and Picardy and replaced the previously used vaccine (MenBVac) [11, 17]. In 2016, in Beaujolais (Auvergne-Rhône-Alpes region), a clonal outbreak caused by a serogroup B cc32 strain was detected (4 cases in less than one month) and a vaccination campaign was implemented for people aged between 2 months and 24 years living, working or studying in the epidemic area [8].

The currently available data do not show any evidence of an impact of Bexsero® on the carriage of serogroup B isolates [15, 18]. Therefore, the objective of the vaccination was to give individual protection [19]. In the vaccination campaign in Côtes-d’Armor, the total vaccination coverage of the targeted 11–19 years-old population was estimated at 43% for the first dose and 34% for the second dose. In the vaccination campaign in Beaujolais, the vaccination coverage was estimated at 44% for 1 dose and 36% for 2 doses for the 12–15 year olds and 14% for 1 dose, 8% for 2 doses for the 16–24 year olds [8]. The vaccination coverages estimated in our campaign are thus quite similar to those observed in the campaign in Beaujolais for the age group the 12–15 years old.

The vaccination coverage in the two schools (84% for 1 dose-79% for 2 doses in the middle school; 56–42% in the high school) was significantly higher compared to vaccination coverage observed in the community (36–27%). Although factors influencing the vaccination coverage rates were not evaluated, several could reasonably be cited. Access to vaccination was easier for children of the two schools as the vaccination sessions were organized directly on-site. Whereas, outside the two schools, people had to consult their general practitioner to get a prescription and go to the pharmacy for each dose, which may have limited access to vaccination. Additionally, the perceived risk of being infected probably varied according to the situation. The perceived risk may be higher for the children attending the same school as the cases, compared to those in the community. Furthermore, in the middle school, the contamination is very likely to have occurred at school whereas in the high school, the two cases were contaminated outside the school during the holidays. Outside the two schools, the perception of the risk may have been even lower. And lastly, the younger the children are, the greater the probable influence of parents in the decision making process concerning vaccination. Other socio-demographic factors might have influenced vaccine uptake [20] such as gender, socio-economic group, education level of parents. However, we had no individual data on the vaccinated population to address this question.

The two sources of data used for estimating vaccination coverage in the community were complementary and the method could be used in future campaigns. Distribution data from wholesale distributors were exhaustive and transmitted regularly during the campaign. However, those data did not contain information about the proportions of each dose. Delivery data obtained from a sample of 18 pharmacies allowed us to estimate the proportions of first and second dose. A limit of the method is that we hypothesized that the proportions of doses 1 and 2 in this sample of pharmacies were the same than in the other pharmacies of the area. The best way to assess vaccination coverage would have been to use the French healthcare reimbursement databases. However, the delay necessary to obtain these data ranges from 6 to 12 months which was not compatible with our objective of real-time monitoring of the vaccination coverage. Although less accurate, our method allowed the health authorities to assess in real time the dynamic of vaccination in the target population.

High variation in the notification rates of adverse effects was observed between the first (31.2%) and the second vaccination campaign (3.7%). This can be explained by the modalities of data collection. Indeed, in the middle school, a reinforced pharmacovigilance follow-up was set up, which led to a higher notification of adverse effects. Whereas, in the second campaign, parents were asked to return the questionnaire only in case of adverse effects, as usual in pharmacovigilance follow-up. This notification rate of adverse effects (3.7%) in the second campaign was in concordance with to that observed in the vaccination campaign in the Beaujolais (3.5%). No severe adverse effects were reported and AE were mostly local reactions. The nature of the reported AE in both campaigns was consistent with the previously described safety profile of the vaccine [21].

## Conclusion

This campaign was successful thanks to the involvement of a large number of professionals from different public health sectors (RHA, French national public health agency, French Ministry of Health, National Reference Centre for meningococci, Pharmacovigilance center, French Ministry of Education, hospitals, Civil protection services, GPs, pharmacists) and provides useful information for future targeted vaccination campaigns.

## Data Availability

The datasets generated and/or analyzed during the current study are not publicly available due to the presence of personal data but anonymized data are available from the corresponding author on reasonable request.

## Abbreviations

IMD: Invasive Meningococcal Disease
RHA: Regional Health Agency
cc: clonal complex
PBT: Protective bactericidal threshold
MATS: meningococcal Antigen Typing System
GP: General Practitioners
AE: Adverse Effects
